# Fingerspelling, signed language, text and picture processing in deaf native signers: The role of the mid-fusiform gyrus

**DOI:** 10.1016/j.neuroimage.2007.01.025

**Published:** 2007-04-15

**Authors:** Dafydd Waters, Ruth Campbell, Cheryl M. Capek, Bencie Woll, Anthony S. David, Philip K. McGuire, Michael J. Brammer, Mairéad MacSweeney

**Affiliations:** a Behavioural and Brain Sciences Unit, Institute of Child Health, University College London, 30 Guilford Street, London WC1N 1EH, UK; b Deafness, Cognition and Language Research Centre, Department of Human Communication Science, University College London, 49 Gordon Square, London WC1H 0PD, UK; c Institute of Psychiatry, King’s College London, De Crespigny Park, London SE5 8AF, UK

## Abstract

In fingerspelling, different hand configurations are used to represent the different letters of the alphabet. Signers use this method of representing written language to fill lexical gaps in a signed language. Using fMRI, we compared cortical networks supporting the perception of fingerspelled, signed, written, and pictorial stimuli in deaf native signers of British Sign Language (BSL). In order to examine the effects of linguistic knowledge, hearing participants who knew neither fingerspelling nor a signed language were also tested. All input forms activated a left fronto-temporal network, including portions of left inferior temporal and mid-fusiform gyri, in both groups. To examine the extent to which activation in this region was influenced by orthographic structure, two contrasts of orthographic and non-orthographic stimuli were made: one using static stimuli (text vs. pictures), the other using dynamic stimuli (fingerspelling vs. signed language). Greater activation in left and right inferior temporal and mid-fusiform gyri was found for pictures than text in both deaf and hearing groups. In the fingerspelling vs. signed language contrast, a significant interaction indicated locations within the left and right mid-fusiform gyri. This showed greater activation for fingerspelling than signed language in deaf but not hearing participants. These results are discussed in light of recent proposals that the mid-fusiform gyrus may act as an integration region, mediating between visual input and higher-order stimulus properties.

## Introduction

Signed languages are natural human languages, distinct from the spoken languages that surround them. Although English is spoken in both the USA and the UK, the signed languages used in these countries (American Sign Language and British Sign Language, respectively) are mutually unintelligible. Signed languages show all the essential characteristics of human language (Hockett, 1968; for discussion, see Sutton-Spence and Woll, 1999) and arise spontaneously wherever there are deaf communities (Sandler et al., 2005; Senghas et al., 2004).

The neural systems that support signed languages have been widely explored using a variety of imaging methods (Bavelier et al., 1998a,b; Emmorey et al., 2002, 2003, 2004, 2005; Levänen et al., 2001; MacSweeney et al., 2002a,b, 2004, 2006; McGuire et al., 1997; Neville et al., 1997, 1998; Petitto et al., 2000; Sakai et al., 2005; Soderfeldt et al., 1994, 1997). These studies reveal that patterns of activation for signed language processing are remarkably similar to those observed for processing spoken languages, with activation observed in left perisylvian regions including inferior frontal and posterior temporal cortices (for review, see Campbell et al., submitted).

**Fig. 1:**
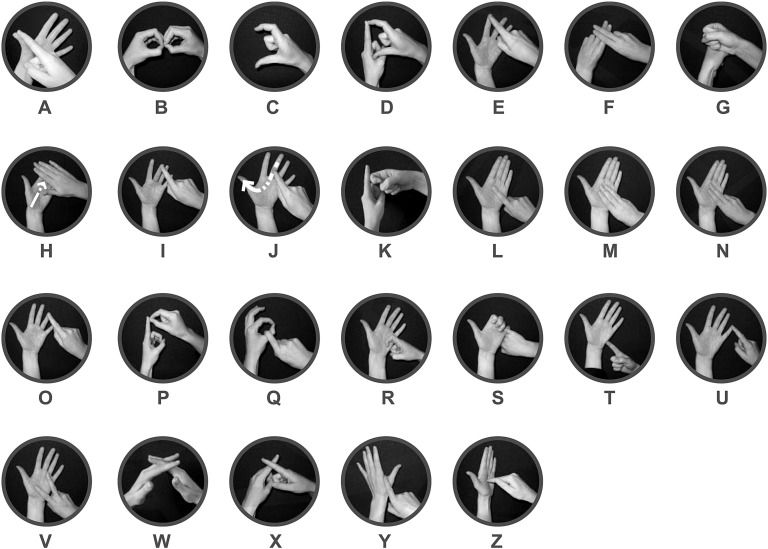
The British manual alphabet. Each letter of the Roman alphabet is represented by a specific hand arrangement. The British manual alphabet differs significantly from more prevalent one-handed varieties such as that used by American signers.

In contrast, although the neural correlates of fingerspelling *production* have been explored (Emmorey et al., 2003), the neural underpinnings of fingerspelling *comprehension* are unknown. Fingerspelling is a manual encoding of written language, used by signers to fill lexical gaps in a signed language (Sutton-Spence and Woll, 1999). British signers use a two-handed variety of fingerspelling in which 26 hand arrangements correspond to the 26 letters of the Roman alphabet (see Fig. 1). Fingerspelling is based on written word forms, which are available to deaf people through reading. Just as a hearing person may spell out an unfamiliar name by uttering the letter names, so can a fingerspeller use the manual alphabet to ‘spell out’ unfamiliar words. Fingerspelling encodes the writing system of a language (a secondary code); therefore, it may be considered a tertiary code (Sutton-Spence, 1994). As such, analogues of fingerspelling are rare, although Braille is similar in this respect.

Fingerspelling shares properties with signed language since both are manually articulated using dynamic action patterns, and both are temporally ordered and rapidly fading. However, since fingerspelling is orthographic, it also shares properties with text. The similarities in motor and perceptual qualities between fingerspelling and signed language, and in representation between fingerspelling and text, suggest that the neural systems that support fingerspelling processing may overlap with those for both signed language and text processing. In the current study, we address this issue by comparing activation patterns observed in deaf native signers of British Sign Language (BSL) while they watched single items presented in four different visual input forms: fingerspelled English words, BSL signs, written English words, and pictures.

This study also allows us to address a current controversy in cognitive neuroscience, specifically: the role of the left mid-fusiform gyrus in processing visual input. The fusiform gyrus within the inferior temporo-occipital regions is activated in a range of visuo-perceptual tasks, and debate currently rages over the nature of these specializations, and the extent to which they should be accounted modular. Processing of faces (Kanwisher et al., 1997) and body parts (Downing et al., 2001) preferentially activate these regions, with distinctive foci having been reported for each stimulus type. A highly specific proposal is that parts of the left mid-fusiform gyrus are specialized for text processing. Cohen et al. have proposed that the left mid-fusiform gyrus should be referred to with the functional label: ‘visual word form area’ (VWFA; Cohen et al., 2000, 2002) because it appears to show a response preference to written words. Throughout this paper, we use the term ‘VWFA’ to refer to the region described by Cohen et al. (2000, 2002) without necessarily subscribing to the position that this portion of the left mid-fusiform gyrus is specialized for visual word form processing (see Discussion).

Cohen et al. (2002) have claimed that an area “subtended by a restricted patch of left-hemispheric fusiform cortex… is reproducibly activated by reading” (p. 1054). They locate the VWFA at the average Talairach coordinates (TC; *x*, *y*, *z*) − 43 − 54 − 12 (Cohen et al., 2000). This proposal is supported by numerous meta-analyses which report consistent activation in this region during reading tasks (Bolger et al., 2005; Fiez and Petersen, 1998; Jobard et al., 2003; Turkeltaub et al., 2002), and further by neuropsychological studies reporting reading deficits in patients with damage to left mid-fusiform cortex (Cohen et al., 2003; Gaillard et al., 2006). Cohen et al. (2002) claim further that the posterior portion of the VWFA (TC *y* = − 43 to − 70) is “unimodal, at least as far as word perception is concerned” (p. 1055), and refer to this as the ‘VWFA proper’. In contrast, they argue that the more anterior region (TC *y* = − 32 to − 54) is less modality specific, responding to both written *and* spoken words.

However, the characterization of the posterior region of the left mid-fusiform gyrus as unimodal and specialized for visual word forms is controversial. Price and Devlin (2003) cite a range of tasks that activate the VWFA in the absence of overt visual word form processing. These include color naming, picture naming, reading Braille, and repeating auditory words. Moreover, picture naming can generate *greater* activation in this region than reading (Bookheimer et al., 1995; Moore and Price, 1999; Price et al., 2006). On this basis, Price and Devlin (2003) argue that this area should be considered neither unimodal nor specialized for visual word form perception. Rather, Devlin et al. (2006) have proposed that this region mediates between abstract visual forms and higher-order stimulus properties such as sound and meaning. For example, hearing participants recruit this region during rhyme judgments in response to pictures (MacSweeney et al., submitted) and auditory words (Booth et al., 2002). Importantly, this hypothesis is not limited to reading; any meaningful, or potentially meaningful, visual stimulus would be expected to activate this region (Devlin et al., 2006).

One way to further illuminate the role of the VWFA is to consider activation in this region when orthographically structured stimuli are presented in different input forms. Tactile perception of orthography, via Braille, has been reported to generate VWFA activation in both late and congenitally blind readers (Büchel et al., 1998). This might suggest, whatever the relative dependence of pictorial or other visual stimuli on the integrity of this area, that the left mid-fusiform gyrus is particularly sensitive to *orthographic* input, even when the orthography is not in a written format. Fingerspelling represents orthography, yet in a very different form to written words or Braille. Fingerspelling allows us to further evaluate the possibility that the left mid-fusiform gyrus is sensitive to orthographically structured input, regardless of input form.

In the study reported here, deaf native signers were presented with lists of meaningful stimuli while fMRI data were collected. Four different visual input forms were investigated: fingerspelled English words (FS), BSL signs (SL), written English words (TEXT), and pictures (PICS). To encourage equivalent semantic coding of every item, participants were required to respond to a sparsely occurring semantically defined target (any animal). These experimental conditions were contrasted with a low-level baseline task, requiring the detection of a color change in a fixation cross.

This design allowed us to address the following questions: [1] In deaf native signers, what are the neural correlates of perceiving FS, SL, TEXT, and PICS? Is there a role for the VWFA in perceiving each of these visual input forms? [2] Does the VWFA show selective specificity for stimuli that are orthographically structured? We addressed this question in two contrasts: first, with static stimuli (TEXT vs. PICS); and second, with visuo-dynamic stimuli (FS vs. SL). In order to dissociate the linguistic and perceptual effects of the FS and SL stimuli, data from hearing non-signers were also included in these analyses. Both signers and non-signers are sensitive to the orthographic structure of TEXT. Therefore, if the VWFA is specialized for TEXT processing, we would predict greater activation in this region for TEXT than for PICS, with no main effect of group and no interaction between visual input form and group. In contrast, signers, but not non-signers, are sensitive to the orthographic structure of FS. If the VWFA is particularly responsive to orthography, regardless of input form, we would expect an interaction in this region between visual input form (FS vs. SL) and language knowledge (signers vs. non-signers), with FS eliciting greater activation than SL in signers but not non-signers.

## Materials and methods

### Participants

All participants were right-handed and without any known neurological or behavioral abnormality. All participants gave informed, written consent to participate in the study, which was approved by the Institute of Psychiatry/South London and Maudsley NHS Trust Research Ethics Committee.

Thirteen (six female) deaf native signers were tested. Their mean age was 27.4 years (range: 18–49 years). All were native signers, having acquired BSL from their deaf parents. All deaf participants reported being born deaf and hearing loss was assessed prior to the scan. This confirmed that all deaf participants were severely or profoundly deaf (80 dB mean loss or greater in the better ear over seven octaves, spanning 125–8000 Hz). Across the group, the mean hearing loss in the better ear was 102 dB. To ensure that all deaf participants had adequate receptive fingerspelling skills, fingerspelling comprehension was tested by showing participants video clips of 25 fingerspelled English words, similar to those used in the fMRI study. Participants then had to sign the word they had seen. Mean level of accuracy was 21/25 (S.D. 2.6). Participants scoring less than 17/25 were not included in the study.

Thirteen (six female) hearing non-signers with no previous exposure to fingerspelling or signed language were also tested. Their mean age was 29.4 years (range: 18–43 years). All were monolingual speakers of English.

All participants were tested on non-verbal IQ (Block Design, WAIS-R), reading ability (NFER-Nelson, 2000; Vernon-Warden, 1996), and English vocabulary (shortened version of the Boston Naming Test; Kaplan et al., 1983). There were no significant differences between deaf and hearing participants in age or non-verbal IQ (*P*-values > 0.1). However, hearing participants scored significantly higher than deaf participants on reading (*P* < 0.001) and English vocabulary (*P* < 0.025). See Table 1 for a summary of participant characteristics.

### Stimuli

**Fig. 2:**
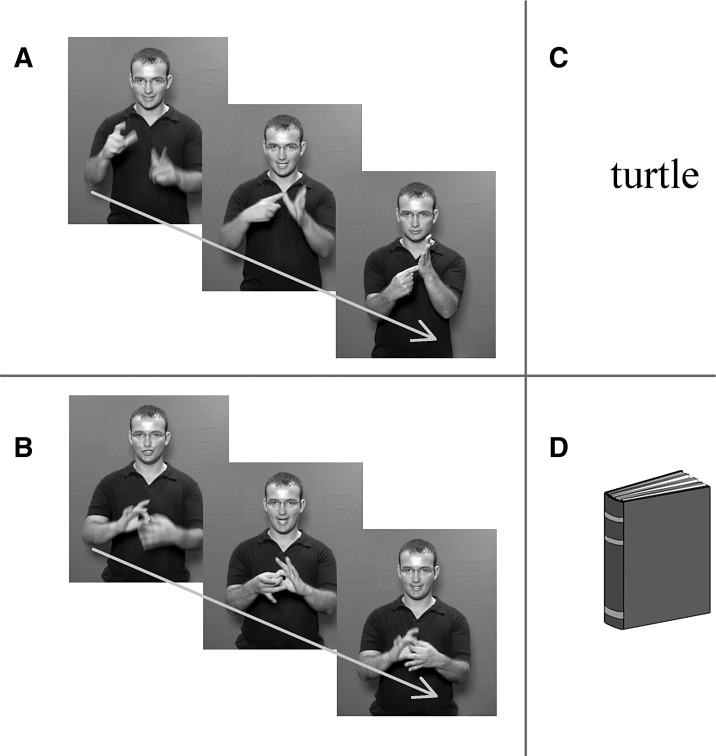
Examples of each type of visual input form. Participants were required to make a button-press response when they saw an animal or animal name in any of the following conditions. (A) Fingerspelling: video stills illustrate each letter of the English word, ‘cat’, as it would be conveyed via fingerspelling. (B) Signed language: three video stills from the BSL sign, ‘chain’. Still images presented here are for illustration purposes only; both fingerspelled and signed stimuli were presented as color, moving video clips. (C) Text: English words were centrally presented in a black, lower-case, serif font. (D) Pictures: taken from the Snodgrass and Vanderwart (1980) normed picture set. Color versions were used in the actual experiment.

One hundred stimulus items were selected. Each item was prepared in four visual input forms: [1] fingerspelled English words (FS); [2] BSL signs (SL); [3] written English words (TEXT); [4] pictures (PICS) (see Fig. 2 for examples). All were concrete nouns, between three and nine letters long. To ensure that the visual properties of the FS and SL stimuli were as similar as possible, only BSL signs articulated with two hands in neutral space in front of the body were selected. Additionally, in most cases we chose signs in which the two hands make contact and handshapes are asymmetrical, as is the case for most fingerspelled letters.

A deaf native signer of BSL modeled the FS and SL stimuli, which were presented as full-color motion video. For both stimulus types, the signer used BSL-appropriate English-derived mouth patterns. The model’s hands came to a rest position between each signed or fingerspelled item. TEXT stimuli were presented in a black, lower-case, serif font, centred against a white background. PICS stimuli were color drawings, the majority of which were color versions of the Snodgrass and Vanderwart (1980) normed picture set.

### Experimental design

Stimuli were presented in alternating blocks of experimental and baseline conditions lasting 30 s and 15 s, respectively. Over the course of the experiment, participants viewed all 100 stimulus items, 25 in each of the four experimental conditions. The presentation of items was counterbalanced such that each participant saw each item in only one visual input form. For example, a participant who saw ‘car’ signed never saw ‘car’ fingerspelled, printed or pictured. Another participant might see ‘car’ fingerspelled, but would never see ‘car’ signed, printed or pictured. This ensured that any differences observed between input forms could not be attributed to semantic differences between stimulus items.

Each stimulus item was viewed twice, with the exception that animal targets were seen only once. Repetitions occurred throughout the run in different blocks of the same input form and were distributed equally across input forms. This sparse repetition was considered unlikely to generate reduced activation due to adaptation to stimulus identity (Grill-Spector et al., 2006).

Ten items were presented in each block. TEXT and PICS were displayed for 2 s, with an interstimulus interval of 1 s. Despite slight variation in the actual duration of individual FS and SL stimuli, the rate of articulation in both these conditions approximates to one item every 3 s. All stimuli were projected onto a screen at the base of the scanner table using a Sanyo XU40 LCD projector, and then projected to a mirror angled above the participant’s head.

Participants were given a target-detection task in all conditions. During the experimental conditions, signers were required to make a button-press response whenever the item was an animal. Non-signers were given the same task for the TEXT and PICS conditions. However, because the FS and SL stimuli were not meaningful for non-signers, they were instead instructed to make a button-press response whenever they identified the start of a FS or SL block. Instructions for non-signers were: “You are going to see blocks of signs, words and pictures. Press the button whenever the word or picture you see is an animal. When you see signs, press the button as soon as you realize that a group of signs has started. Please always look at the signer’s face. Don’t try to follow the signer’s hands around the screen.” These instructions were designed to optimize matching of viewing behavior between signers and non-signers, since it has been established that during natural signed discourse, signers fixate the interlocutor’s face and do not show saccades to hand actions (Agrafiotis et al., 2003; Muir and Richardson, 2005).

During the baseline condition, a black cross-hair set against a white background appeared in the centre of the screen. Participants made a button-press response when the cross-hair turned red. Targets in both the experimental and baseline conditions occurred at random positions throughout the block. All participants practiced the tasks outside the scanner with materials not used in the actual experiment.

### Imaging parameters

Gradient echoplanar fMRI data were acquired with a 1.5-T General Electric Signa Excite scanner (Milwaukee, WI, USA), fitted with TwinSpeed gradients and an 8-channel quadrature head coil. Three hundred T_2_*-weighted images depicting BOLD contrast were acquired at each of 40 near-axial 3 mm thick planes parallel to the intercommissural (AC–PC) line: 0.3 mm interslice gap; TR = 3 s, TE = 40 ms, flip angle = 90°. The field of view for the fMRI runs was 240 mm, and the matrix size was 64 × 64, with a resultant in-plane voxel size of 3.75 mm. High-resolution structural scans were acquired for registration of individual fMRI datasets to Talairach space (Talairach and Tournoux, 1988). These comprised 40 near-axial 3 mm slices (0.3 mm interslice gap), which were acquired parallel to the AC–PC line (TR = 3 s, TE = 40 ms, flip angle = 90°). The field of view for the structural runs was 240 mm, and the matrix size was 128 × 128, with a resultant in-plane voxel size of 1.875 mm.

### Data analysis

fMRI data were first corrected for motion artifact. The mean intensity at each voxel was computed over all time points, resulting in an average image volume. The image volumes at each time-point were then registered to this average using rigid body transformation (*x*, *y* and *z* rotation and translation) by maximization of correlation. Maximum displacements in the *x*-, *y*- and *z*-dimensions over all intra-cerebral voxels were computed for each participant using the estimation of rigid body motion. The mean values of these displacements [s.d.] in each group were: Deaf group: *x* = 1.4 mm [0.7]; *y* = 2.8 mm [2.1]; *z* = 7.2 mm [4.8]; Hearing group: *x* = 1.5 mm [1.3]; *y* = 2.6 mm [1.6]; *z* = 6.7 mm [4.4]. There were no significant group differences in any of the three dimensions (*t*-values: 0.23, 0.22 and 0.23, respectively; all *P*-values > 0.1).

Data were then smoothed using a Gaussian filter (FWHM 7.2 mm). The least-squares fit was computed between the observed time series at each voxel and the convolutions of two gamma variate functions (peak responses at 4 s and 8 s) with the experimental design (Friston et al., 1998). The best fit between the weighted sum of these convolutions and the time series at each voxel was computed using the constrained BOLD effect model suggested by Friman et al. (2003). This computation constrained the range of fits to those that reflect the physiological features of the BOLD response. Following computation of the model fit, a goodness of fit statistic was derived by calculating the ratio between the sum of squares due to the model fit and the residual sum of squares (SSQ ratio) at each voxel. The data were detrended to remove low-frequency noise and permuted using the wavelet-based method described by Bullmore et al. (2001). Significant SSQ values were identified by comparing this statistic with the null distribution, determined by repeating the fitting procedure 20 times at each voxel. This procedure preserves the noise characteristics of the time-series during the permutation process and provides good control of Type-I errors. The voxelwise SSQ ratios were calculated for each participant from the observed data and, following time series permutation, were transformed into Talairach and Tournoux’s (1998) standard space using the following procedure (Brammer et al., 1997; Bullmore et al., 1996).

At the Talairach transformation stage, inversion recovery and T_2_*-weighted Talairach templates were constructed from data from 20 (10 female) healthy controls aged 22 to 55. These data were obtained at our own imaging centre. The images for each individual were transformed to Talairach space manually with AFNI, which uses manual landmark identification. The positions of morphological landmarks were checked following the transformation. To ensure compatibility and generalizability of findings, morphological correspondence to Talairach templates used in other image packages was also checked. Once the template was constructed, it was used as the target for automatic spatial normalization, which was performed in two stages. First, the fMRI data were transformed to high-resolution T_2_*-weighted image of each participant’s own brain using a rigid body transform. Second, an affine transformation to the Talairach template was computed. The cost function for both transformations was the maximization of correlation between the images. Voxel size in Talairach space was 3 × 3 × 3 mm.

### Group analysis

Identification of active 3-D clusters was performed by thresholding the median voxel-level SSQ ratio maps at the false positive probability of 0.0025. The activated voxels were assembled into 3-D connected clusters and the sum of the SSQ ratios (statistical cluster mass) determined for each cluster. This procedure was repeated for the median SSQ ratio maps, obtained from the wavelet-permuted data, to compute the null distribution of statistical cluster masses under the null hypothesis. The clusterwise false positive threshold was then set using this distribution to give an expected false positive rate of less than one cluster per brain (Bullmore et al., 1999).

In the case of one large, contiguous cluster (elicited by FS stimuli in deaf native signers), the output from the cluster analysis was further analyzed using ‘MClust’, a model-based cluster analysis package (Fraley and Raftery, 2002, 2003). This package uses the Bayesian Information Criterion to determine the number of clusters in an input data set using a hierarchical Expectation Maximization clustering algorithm. The Talairach coordinates and amplitude of the BOLD signal at each activated voxel were used as input to MClust. The main reason for using model-based clustering was to estimate the number/size of foci subsumed within spatially contiguous clusters without incurring the loss of power that would occur at higher statistical thresholds.

### ANOVA

Differences between experimental conditions were calculated by fitting the data at each voxel in which all participants had non-zero data using the following linear model, *Y* = *a* + *bX* + *e*, where *Y* is the vector of BOLD effect sizes for each individual, *X* is the contrast matrix for the particular inter-condition/group contrasts required, *a* is the mean effect across all individuals in the various conditions/groups, *b* is the computed group/condition difference, and *e* is a vector of residual errors. To reduce outlier effects, the model was fitted by minimizing the sum of absolute deviations, rather than the sums of squares. The null distribution of *b* was computed by permuting data between conditions (assuming the null hypothesis of no effect of experimental condition) and refitting the above model. Group difference maps were computed as described above at the voxel or cluster level by appropriate thresholding of the null distribution of *b*.

### ANCOVA

Analysis of covariance was used to address behavioral differences in reaction time to FS and SL stimuli in deaf participants in relation to the patterns of activation (see Results, below). Differences in responses (*R*) were inferred at each voxel using the linear model, *R* = *a*0 + *a*1*H* + *a*2*X* + *e*, where *H* codes the contrast(s) of interest between groups or conditions, *X* is a covariate and *e* is the residual error. Maps of the standardized coefficient (size of condition or group difference; *a*1) were tested for significance against the null distribution of *a*1 (no effect of group membership or condition) generated by repeatedly refitting the above model at each voxel following randomization of group or condition membership (*H*). These analyses were performed at the second level, using mean reaction times as input.

### Defining the range of the visual word form area (VWFA) and its right hemisphere homologue

Given the problems inherent in using functional localizers (see Friston et al., 2006; Friston and Henson, 2006), for the purposes of the current study we defined the location of the VWFA on the basis of previously published data. Cohen et al. (2000) report coordinates from a group analysis (*n* = 5; TC − 42, − 57, − 6; S.D. = ∼ 5 mm for all dimensions) and the average coordinates from analyses of individual participants (*n* = 5; TC − 43, − 54, − 12; S.D. = ∼ 5 mm for all dimensions). By taking both of these datasets into account, we define the approximate location of the VWFA in the *x*- and *z*-dimensions as: TC *x* = − 37 to − 48 and TC *z* = − 1 to − 17. In the *y*-dimension, the coordinates reported by Cohen et al. (2000) for the ‘VWFA proper’ (in contrast to those for multimodal regions) are used to constrain our area of interest (TC *y* = − 43 to − 70). Therefore the range we use in the current paper to define the VWFA is: TC *x* = − 37 to − 48, TC *y* = − 43 to − 70, TC *z* = − 1 to − 17. Studies referring to the right hemisphere homologue of the VWFA (e.g., Cohen et al., 2003, 2004) describe it as symmetrical to the VWFA in the left hemisphere. Thus, we use the corresponding right hemisphere coordinates (TC *x* = 37 to 48, TC *y* = − 43 to − 70, TC *z* = − 1 to − 17) to define the right hemisphere homologue of the VWFA. Given that these ranges are approximate, and given the spatial inaccuracies inherent in localization of fMRI data, where only one coordinate of a set of three (*x*, *y* or *z*) was outside this range by 1 mm, we included this as being within the range of the VWFA or its right hemisphere homologue.

## Results

### Behavioral data

Mean accuracy and reaction time (RT) data for each task and for each group are shown in Table 2. To reflect the planned contrasts applied to the fMRI data, the static (TEXT/PICS) and dynamic (FS/SL) conditions were analyzed separately.

Both deaf and hearing participants performed the animal detection task during the TEXT and PICS conditions. An ANOVA conducted on the accuracy data, with Visual Input Form (TEXT/PICS) as the within-subjects factor and Group (deaf/hearing) as the between-subjects factor, showed no main effects of Visual Input Form or Group and no interaction (all *P*-values > 0.1). With regard to the RT data, there was no main effect of Group and no interaction. However, there was a significant main effect of Visual Input Form, *F*(1,24) = 11.3, *P* < 0.005, indicating that both groups were quicker to respond to PICS than TEXT (see Table 2).

Deaf participants were equally accurate on the FS and SL tasks (*P* > 0.1), however they were significantly slower at making FS than SL judgments, *t*(12) = 5.1, *P* < 0.001. Since the hearing participants did not know BSL, they were simply asked to press a button at the beginning of each FS/SL block. Therefore, no behavioral data are reported for the hearing group during the SL and FS conditions.

Finally, there were no significant differences in accuracy or RT between deaf and hearing groups on the baseline (target detection) task (all *P*-values > 0.1).

Following the scan, all signing participants performed a comprehension test of the FS and SL items presented in the scanner. All signing participants showed a high level of comprehension: FS mean level of accuracy was 23/25 (S.D. 1.8); SL mean level of accuracy was 24/25 (S.D. 0.9).

### fMRI data

#### Activation for each input form relative to baseline in deaf native signers (see Fig. 3/Table 3)

##### Fingerspelling (FS) greater than baseline

**Fig. 3:**
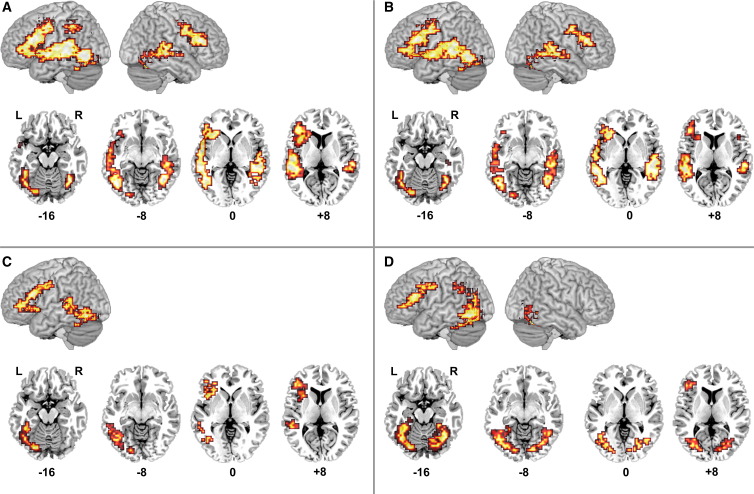
Activation for each visual input form relative to the baseline task in deaf native signers (*n* = 13; voxelwise *P* = 0.05; clusterwise *P* = 0.0025). (A) FS > baseline; (B) SL > baseline; (C) TEXT > baseline; (D) PICS > baseline. On lateral renderings, activated voxels up to 15 mm beneath the cortical surface are displayed. Contiguous axial slices from TC *z* = − 16 to 8 mm are also shown. L = left. R = right.

FS elicited significant activation in a large portion of the left hemisphere (number of voxels = 1298/350.46 cm^3^). This activation included parts of the occipital, temporal and frontal lobes. A model-driven cluster analysis (MClust; see Materials and methods section) was used to decompose the different regions incorporated in this large cluster. This analysis revealed six distinct areas of activation located in: [1] the anterior cingulate; [2,3] the left frontal cortex, including the precentral gyrus, and the inferior, middle and superior frontal gyri; [4,5] the left inferior and middle, and the superior temporal gyri; and [6] the left fusiform gyrus. The focus of this left fusiform activation corresponded with the coordinates of the VWFA (see Fig. 3A/Table 3; see Materials and methods for details regarding our definition of the VWFA).

FS also engaged the left precuneus, and the right occipito-temporal and frontal cortices. Activation in the left precuneus extended to the postcentral gyrus and the inferior and superior parietal lobules. Activation in right occipito-temporal cortices included the right fusiform gyrus (including the right hemisphere homologue of the VWFA; see Fig. 3A/Table 3), and extended to the inferior, middle and superior temporal gyri. Finally, right frontal lobe activation included the precentral gyrus, and the inferior and middle frontal gyri.

##### Signed language (SL) greater than baseline

SL generated significant activation in four regions: [1,2] the left and right inferior and middle frontal gyri, extending to the precentral gyri; and [3,4] the left and right fusiform and inferior temporal gyri, with foci consistent with the location of the VWFA and its right hemisphere homologue (see Fig. 3B/Table 3). The left temporal activation extended from the lingual and fusiform gyri to the inferior, middle and superior temporal gyri. Except for activation in the lingual gyrus, a similar pattern of activation was observed in right temporal cortices.

##### Written English words (TEXT) greater than baseline

Two left hemisphere regions were engaged during the TEXT task: [1] the left fusiform gyrus; and [2] the left precentral and inferior/middle frontal gyri. Activation in the left fusiform gyrus extended posteriorly to the left lingual gyrus, and also superiorly to the posterior and middle portions of the left inferior, middle and superior temporal gyri. Although the focus of this activation was not located within the range of the VWFA (see Fig. 3C/Table 3), this cluster nevertheless included voxels in this region (number of voxels = 74/19.98 cm^3^; TC *x* = − 40 to − 47; TC *y* = − 52 to − 67; TC *z* = − 16 to 0).

##### Pictures (PICS) greater than baseline

Three regions were engaged during the PICS task: [1,2] the left and right fusiform/inferior temporal gyri; and [3] the left inferior/middle frontal gyri extending to the precentral gyrus. Activation in the left fusiform gyrus extended inferiorly to include superior portions of the left cerebellum. It also extended anteriorly and superiorly to the left middle occipital gyrus, the left inferior and middle temporal gyri, the left precuneus, the left cuneus, and the left inferior and superior parietal lobules. This cluster included the VWFA (see Fig. 3D/Table 3). Similarly, right fusiform gyrus activation extended inferiorly to include superior portions of the right cerebellum, and anteriorly and superiorly to the middle occipital gyrus and the cuneus. This cluster included activation in the right hemisphere homologue of the VWFA (see Fig. 3D/Table 3).

Since hearing non-signers are not the focus of this paper, the 3-D maxima for this group for each input form are reported in Table 4, but are not described in detail. A figure illustrating activation for each visual input form relative to the baseline task in hearing non-signers appears as online supplementary material.

##### Orthographic vs. non-orthographic static stimuli: TEXT vs. PICS

To investigate the role of orthographic structure when processing *static* stimuli available for semantic processing to both groups, a mixed-model ANOVA was conducted with Visual Input Form (TEXT/PICS) as the within-subjects factor and Group (deaf native signers/hearing non-signers) as the between-subjects factor (voxelwise *P* = 0.05; clusterwise *P* = 0.005).

**Fig. 4:**
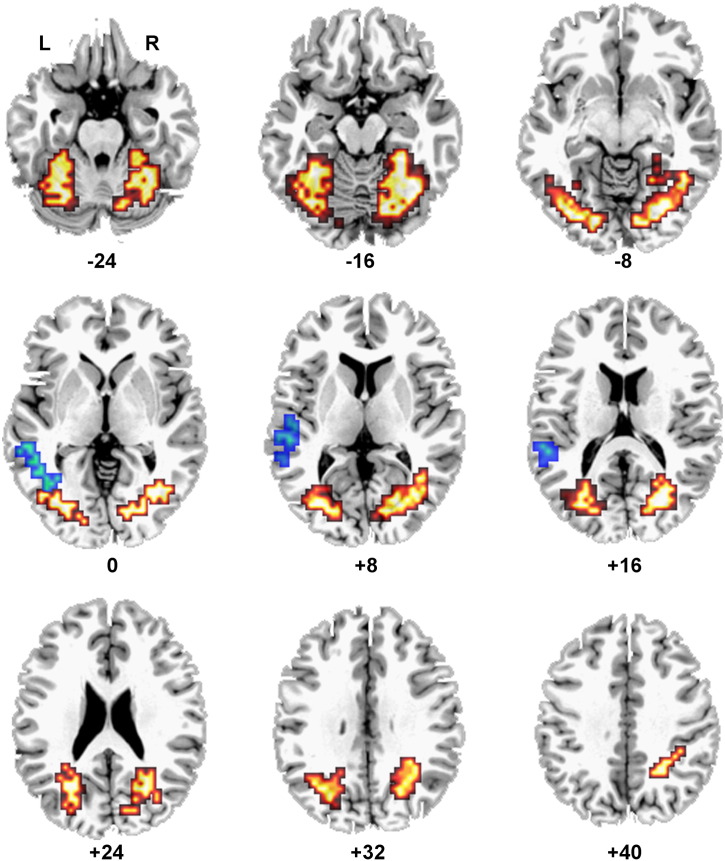
Orthographic and non-orthographic static stimuli (voxelwise *P* = 0.05; clusterwise *P* = 0.005). Main effect of Visual Input Form (TEXT vs. PICS) across deaf and hearing participants. Blue voxels represent the location of greater activation for TEXT than PICS. Orange/yellow voxels represent the location of greater activation for PICS than TEXT. Contiguous axial slices from TC *z* = − 24 to 40 mm are shown. L = left. R = right.

As predicted, there was no significant main effect of Group and no interaction, but there was a significant main effect of Visual Input Form (see Fig. 4/Table 5). TEXT engaged the left middle and superior temporal gyri to a greater extent than PICS. In contrast, there was significantly greater activation for PICS than TEXT in the left middle occipital/fusiform gyri and the right cerebellum, extending to the right fusiform and inferior temporal gyri. Activation within the left hemisphere extended from the lingual/fusiform gyri anteriorly and superiorly to the middle occipital gyrus, the precuneus and the cuneus (see Fig. 4). In the left hemisphere, only 69 of the 410 activated voxels within this cluster (18.63 of 110.70 cm^3^) fell within the range of the VWFA (range: TC *x* = − 36 to − 43; TC *y* = − 63 to − 67; TC *z* = − 10 to − 13). In the right hemisphere, just 168 of 524 activated voxels (45.36 of 141.48 cm^3^) were located at coordinates consistent with the right hemisphere homologue of the VWFA (range: TC *x* = 36; TC *y* = − 63 to − 70; TC *z* = − 13 to − 3).

##### Orthographic vs. non-orthographic visuo-dynamic stimuli: FS vs. SL

To investigate the role of orthographic structure when processing *visuo-dynamic* stimuli, and to examine the effect of language knowledge, a mixed-model ANOVA was conducted with Group (deaf native signers/hearing non-signers) as the between-subjects factor and Visual Input Form (FS/SL) as the within-subjects factor (voxelwise *P* = 0.05; clusterwise *P* = 0.005).

**Fig. 5:**
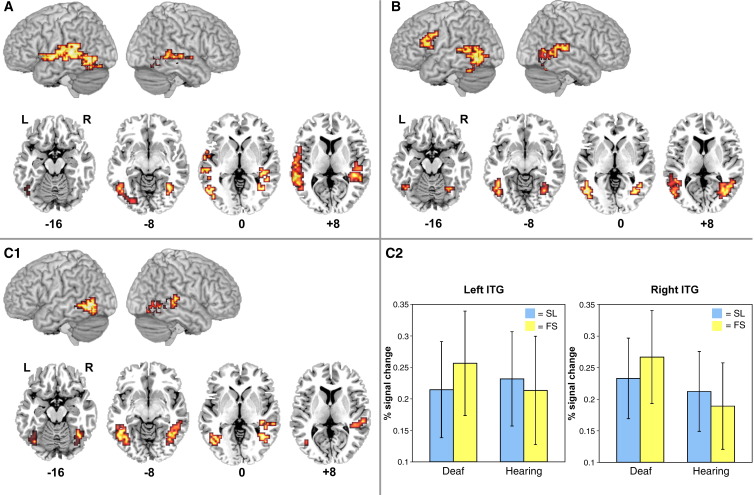
Orthographic and non-orthographic visuo-dynamic stimuli (voxelwise *P* = 0.05; clusterwise *P* = 0.005). (A) Regions activated more by deaf than hearing participants across both FS and SL stimuli are shown. No regions were activated more by hearing than deaf participants. (B) Regions activated more by FS than SL stimuli across both deaf and hearing groups are shown. No regions were activated more by SL than FS. (C1) There was a significant interaction between Group and Visual Input Form in the left and right inferior temporal gyri. (C2) Graphs show percent BOLD signal change in each condition and in each group. Error bars represent standard deviation. ITG = inferior temporal gyrus. On lateral renderings, activated voxels up to 15 mm beneath the cortical surface are displayed. Contiguous axial slices from TC *z* = − 16 to 8 mm are also shown. L = left. R = right.

The main effect of Group indicated that deaf native signers engaged the left and right fusiform/inferior temporal gyri to a greater extent than hearing non-signers (see Fig. 5A/Table 6). Activation was focused within the range of the VWFA and its right hemisphere homologue, and extended anteriorly and superiorly to the middle and superior temporal gyri in both left and right hemispheres. There were no regions in which non-signers showed greater activation than signers.

The main effect of Visual Input Form indicated three regions of greater activation for FS than SL across both groups (see Fig. 5B/Table 6): [1,2] the left and right fusiform/inferior temporal gyri; and [3] the left inferior/middle frontal gyri, extending to precentral gyrus. Foci for activated clusters in the left and right fusiform gyrus were consistent with the VWFA and its right hemisphere homologue. Both left and right hemisphere activations extended anteriorly and superiorly to the middle and superior temporal gyri. There were no regions in which activation was greater for SL than FS.

As predicted, a significant Visual Input Form × Group interaction was identified in the left and right posterior inferior temporal gyri (see Fig. 5C/Table 6). This interaction was due to greater activation during FS than SL perception in the deaf group, but not in the hearing group, who knew neither fingerspelling nor sign. The foci of these activated clusters were within the boundaries of the VWFA and its right hemisphere homologue. In the left hemisphere, 75 of 97 activated voxels (20.25 of 26.19 cm^3^) fell within the VWFA (range: TC *x* = − 36 to − 47; TC *y* = − 63 to − 70; TC *z* = − 10 to 0). The remaining 22 voxels (5.94 cm^3^) were located within the defined *x* and *y* range, but were located slightly more superiorly (up to TC *z* = 10). In the right hemisphere, 65 of 123 activated voxels (17.55 of 33.21 cm^3^) were located within the right hemisphere homologue of the VWFA (range: TC *x* = 40 to 43; TC *y* = − 52 to − 63; TC *z* = − 13 to 0). The remaining right hemisphere voxels extended from the fusiform gyrus to the middle and superior temporal gyri.

##### FS vs. SL in deaf native signers only

The significant interaction reported above suggests that deaf native signers recruit portions of the fusiform gyri to a greater extent than hearing non-signers, especially during the perception of FS in contrast to SL stimuli. Since deaf and hearing participants, of necessity, performed different tasks during the perception of FS and SL stimuli, the robustness of this finding was further assessed by examining the contrast between FS and SL stimuli in deaf native signers alone (voxelwise *P* = 0.05; clusterwise *P* = 0.005). FS generated greater activation than SL in left and right middle and inferior occipito-temporal cortices, and in the left inferior frontal gyrus (see Table 7). In contrast, there were no regions in which SL elicited greater activation than FS.

To address the concern that this finding may be related to slower reaction times to FS than SL stimuli in deaf native signers (see Table 2), this analysis was repeated, including reaction time as a covariate (voxelwise *P* = 0.05; clusterwise *P* = 0.005). Again, the left and right mid-fusiform gyri were recruited to a greater extent by FS than SL stimuli (see Table 7).

Finally, in hearing participants, substantial individual variability has been reported in the precise location of fusiform activation in response to orthographic stimuli (e.g., Polk et al., 2002). We therefore examined data from each of the 13 deaf native signers individually when FS was contrasted with SL (voxelwise *P* = 0.05; clusterwise *P* = 0.01; no covariate). The results of this analysis support the group analysis reported above. Ten out of 13 signers generated greater activation for FS than SL within the range of the VWFA, while 12 of 13 signers generated greater activation for FS than SL within the range of its right hemisphere homologue (see Table 8). In summary, these analyses suggest a special role for the left and right occipito-temporal cortices when deaf native signers process FS relative to SL stimuli.

## Discussion

Here we identify, for the first time, the neural network supporting the perception of fingerspelling (FS), a manual representation of orthography, in deaf native signers. The network recruited during FS perception was very similar to that for the perception of single signs (SL). Both FS and SL engaged a fronto-temporal network in both hemispheres. This included the inferior and middle frontal gyri, the precentral gyri, the fusiform gyri, and the inferior, middle and superior temporal gyri. Activation was more extensive in the left than right hemisphere, incorporating more regions in the frontal and parietal lobes.

A specific aim of this study was to contrast orthographic and non-orthographic stimuli. Accordingly, the involvement of the mid-fusiform gyrus, the region of the proposed ‘visual word form area’ (VWFA) (Cohen et al., 2000, 2002; Dehaene et al., 2002, 2005), in processing FS, SL, written words (TEXT) and pictures (PICS) was of particular interest.

Three main findings are relevant to current proposals regarding the role of this region. First, both deaf native signers and hearing non-signers engaged the left mid-fusiform gyrus in response to all four visual input forms, even though neither SL nor PICS are orthographically structured, and hearing non-signers were naïve to the meaning of the FS and SL stimuli. Thus, these data support the view that the left mid-fusiform gyrus is not *restricted* to processing visual word forms (Price and Devlin, 2003). However, Cohen and Dehaene do not propose that this region is restricted to processing such forms, but rather that it shows a *response preference* to visual word forms (Cohen and Dehaene, 2004).

However, our second main finding does not appear to support this response preference proposal (Cohen and Dehaene, 2004), since neither group engaged the left mid-fusiform gyrus to a greater extent during TEXT than PICS processing. Rather, and in accordance with previous studies of reading and picture naming (Bookheimer et al., 1995; Moore and Price, 1999; Price et al., 2006), greater activation was observed for processing TEXT than PICS in the left middle/superior temporal gyri, a region known to play an important role in phonological processing during word reading (e.g., Turkeltaub et al., 2003). In contrast, PICS activated portions of the VWFA and surrounding regions more than TEXT. Only a small proportion of this differential activation was restricted to the range of the VWFA or its right hemisphere homologue. Activation extended beyond the left and right mid-fusiform gyri, expanding superiorly and posteriorly from the lingual and fusiform gyri to the inferior and middle occipital gyri, the precuneus, and the cuneus. Nevertheless, these data are consistent with reports of greater activation for picture naming than word reading in the left mid-fusiform gyrus and surrounding cortex (Price and Devlin, 2003; Price et al., 2006). Therefore, these data appear to be inconsistent with a strong version of Cohen and Dehaene’s (2004) proposal that visual word forms preferentially activate the left mid-fusiform gyrus.

Nevertheless, the third finding from the current study may lend some support to a modified version of the VWFA hypothesis: a significant interaction identified regions of the left and right mid-fusiform gyri that were engaged to a greater extent during FS than SL processing by deaf, but not hearing, participants. The location of this interaction was predominantly restricted to the VWFA and its right hemisphere homologue. Analysis of the deaf group alone, and inspection of the individual participant data, supported this finding. Further analyses of covariance suggested that this effect was not related to slower reaction times to FS than SL stimuli. Deaf participants were equally accurate on both the FS and SL in-scanner tasks. Therefore, it is unlikely that difficulty accounted for the reaction time differences between conditions. Both FS and SL are delivered over time. Signs, however, are limited to one or two movements. In contrast, the number of movements in a fingerspelling is related to the number of letters in the corresponding written word. Thus, a later uniqueness point for FS than SL stimuli is most likely to account for the reaction time differences observed. Finally, the involvement of the right mid-fusiform gyrus in this interaction is not unexpected. It has been argued on the basis of data from healthy readers (Cohen et al., 2002, 2003; Dehaene et al., 2002), and also on the basis of neuropsychological data (Hillis et al., 2005), that the right hemisphere homologue of the VWFA may play an ancillary role in processing orthographically structured input.

To summarize, the present study presents three main findings relevant to the debate regarding the role of the mid-fusiform gyrus: [1] both deaf and hearing groups engaged the left mid-fusiform gyrus in response to all four visual input forms; [2] neither group activated the left mid-fusiform gyrus more for processing TEXT than PICS; [3] deaf participants recruited portions of the left and right mid-fusiform gyri to a greater extent during FS than SL processing. Although these findings appear contradictory, we argue that they can be accommodated within the recent account of the role of the left mid-fusiform gyrus proposed by Devlin et al. (2006), with one small modification. On the basis of a visual masked priming study manipulating phonology and semantics, Devlin et al. propose that the left mid-fusiform gyrus acts as an interface between incoming visual information and higher-order stimulus properties, such as sound (phonology) and meaning (semantics) (see also Xue et al., 2006). Since the left mid-fusiform gyrus is thought to form a continuation of the ventral visual processing stream, specialized for extracting basic properties from static visual stimuli (e.g., DeYoe and Van Essen, 1988), this proposal is currently thought to apply only to static visual input (e.g., words and pictures). In contrast, visuo-dynamic stimuli are thought to be processed predominantly within a dorsal processing stream, extending into more superior regions of the temporal lobe (Ungerleider and Mishkin, 1982). However, it has recently been demonstrated that dynamic stimuli may also be processed within the ventral stream (e.g., Beauchamp et al., 2002; Grossman and Blake, 2002). Extending Devlin et al.’s (2006) hypothesis to suggest that the left mid-fusiform gyrus is also involved in mapping *visuo-dynamic* input to higher-order representations allows us to accommodate the current data regarding fingerspelling perception within their framework. Each of the three main findings outlined above shall be considered in terms of mapping or integration demands between the incoming stimulus and its higher-order stimulus properties such as orthography, phonology, and semantics.

### 1) Deaf and hearing participants engaged the left mid-fusiform gyrus during the perception of all stimulus types

Since all stimuli were meaningful for deaf participants, it can be argued that perception of each stimulus type, both static and dynamic, involved mapping to higher-order stimulus properties. We cannot establish from the current data whether these may be orthographic, phonological (mapping to either sign- or speech-based phonology) or semantic. An important question for future research is to determine which of these properties is more heavily weighted for each input type.

Hearing participants had no knowledge of FS or SL, yet they also activated the mid-fusiform gyrus during the perception of these stimuli. One possibility is that hearing participants attempted to map the FS and SL input to meaning although this was not required. Hearing participants *were* required to perform the semantic detection task during the TEXT and PICS blocks. We have previously shown that when input is not understood by participants (e.g., non-signers watching signs) and they are asked to guess which stimulus may be semantically anomalous, activation is generated similar to that observed for actual semantic processing, including activation in portions of the left and right fusiform gyrus (MacSweeney et al., 2004). A similar explanation may account for mid-fusiform activation in hearing participants viewing the FS and SL stimuli in the present study.

### 2) Greater activation in left and right mid-fusiform gyri and surrounding regions for PICS than TEXT

Words are perceived almost entirely on the basis of their form, while pictures require processing of form, depth and color: thus involving greater visual processing demands (see Devlin et al., 2006). Although our participants were slower to respond to TEXT than PICS, it has been argued that reaction time is not always proportionate to strength of activation observed in fMRI studies (Henson, 2003). Therefore, the greater visual integration demands of the PICS than TEXT stimuli may account for greater activation for PICS than TEXT in the vicinity of the VWFA. This may be particularly relevant to the current study since black text was contrasted with color pictures. In addition, it could be argued that PICS have the potential to integrate with *more* higher-level information than TEXT, since TEXT, but not PICS, already provides the orthographic representation.

### 3) Greater activation for FS than SL in left and right mid-fusiform gyri in signers

A significant interaction identified portions of the left and right mid-fusiform gyri that were engaged to a greater extent during FS than SL processing in deaf native signers but not hearing non-signers. This interaction was essentially restricted to the VWFA and its right hemisphere homologue. As noted above, fingerspelled English words may be more visually complex than the simple noun-signs presented in this study. Yet, we argue that such perceptual differences are unlikely to account for differential activation in the deaf group, since no such difference was observed in hearing participants, who were both fingerspelling- and sign-naïve. However, it is still possible that attentional demands differed between signers and non-signers since, by necessity, the two groups performed different tasks during the FS and SL conditions. A further study in which both groups perform a non-linguistic task while viewing these stimuli may address this concern.

One interpretation of our finding that deaf native signers engaged portions of the VWFA and its right hemisphere homologue more for FS than SL stimuli is that this region is involved in mapping orthographically structured input, regardless of input form, to corresponding visual word forms. This may be viewed as a modification of the Cohen and Dehaene (2004) position regarding the role of this area. This region is activated with remarkable consistency by different types of scripts (e.g., alphabetic, syllabic, logographic) (Bolger et al., 2005). Furthermore, activation has been reported in the left mid-fusiform gyrus during spelling judgments in response to *auditorily* presented words (Booth et al., 2002) and in late blind participants, when reading words using Braille (Büchel et al., 1998). Even when the orthographic input is visuo-dynamic, as in the current study, the mid-fusiform gyrus is engaged. Thus, it could be argued that the left mid-fusiform gyrus is involved in mapping *orthographic* input (FS but not SL) to word forms, and that the modality (auditory/tactile/visual) of the input is not critical.

Additional arguments regarding integration with representations other than those that are orthographic, as suggested by Devlin et al. (2006), can also be made. Since the deaf participants in the current study were native signers, both FS and SL input may be mapped to *sign* phonology (i.e., the sublexical components of signs (Stokoe, 1960; for reviews, see Brentari, 1998; Sandler and Lillo-Martin, 2006)). It is also likely that deaf native signers map FS input to *speech* phonology: [1] fingerspelling does not represent a signed language; rather, fingerspelling manually encodes the written form of a *spoken* language; [2] up to 99% of fingerspelled words occurring in natural signed discourse in British signers are accompanied by an English-derived mouth pattern (Sutton-Spence and Day, 1997); thus, the motoric/visemic component of speech phonology is reliably present in the production/perception of FS; (3) most signers are to some degree bilingual in speech and sign and so have some knowledge of the phonological structure of speech (MacSweeney et al., submitted). It is possible, therefore, that signers map FS input to a wider range of higher-level representations than SL input. For FS input, mappings are likely to include those based on orthographic, semantic, and sign- and speech-based phonological information. In contrast, while SL input is likely to be mapped to semantics and sign-based phonological representations, it is much less likely to be integrated with orthography or speech-based phonological representations. Signed languages do not have an orthographic representation and English orthography is not encoded in signs. With regard to speech phonology, although signed nouns often occur with an English-derived mouth pattern in fluent BSL discourse (Sutton-Spence and Day, 2001), this is to a lesser degree than for FS.

### Other functional specializations of inferior temporo-occipital regions

So far, we have discussed our findings only in the context of current controversies regarding the functional specialization of the proposed Visual Word Form Area (VWFA; Cohen et al., 2000, 2002). However, portions of inferior temporal cortex located very close to the VWFA (Downing et al., 2006; Reddy and Kanwisher, 2006; Spiridon et al., 2006) have also been shown to be specialized for processing faces (fusiform face area (FFA); Kanwisher et al., 1997) and bodies (extrastriate body area (EBA); Downing et al., 2001). Since faces and bodies are present in both the FS and SL stimuli, it is not surprising that in comparison to a fixation baseline both FS and SL generated activation in left and right inferior temporal cortices in both groups. It is also possible that the appearance of a number of faces, body parts and animals in the PICS condition may be relevant to our finding of greater activation for PICS than TEXT in left and right mid-fusiform gyri.

Since faces and bodies were equally present in both the FS and SL stimuli, we did not form *a priori* hypotheses relating FFA or EBA to any differential activation we might find. Nevertheless, it may be that *attention* to faces and bodies differed across the two conditions, in particular for signers, who understood both stimulus types. Since it has been shown that the EBA responds strongly to hands (Downing et al., 2001), one possibility is that the greater visual complexity of the FS stimuli may have driven greater attention to the hands during the FS condition. Eye-tracking research, however, reports no difference in native signers’ viewing behavior for fingerspelling and signed language (Agrafiotis et al., 2003; Muir and Richardson, 2005). Future research using functional localizers to identify the location of the FFA, EBA and VWFA, and that includes faces and bodies in a baseline condition, would help to clarify the involvement of these different but somewhat overlapping functional areas in viewing FS and SL stimuli. Furthermore, such studies may help to arbitrate between accounts of inferior temporal cortex rooted in the notion of functionally distinct regions (e.g., Downing et al., 2001; Kanwisher et al., 1997) and accounts couched in terms of distributed object representations (e.g., Haxby et al., 2001).

In summary, our data suggest a special role for the left and right mid-fusiform gyri in processing fingerspelling, particularly in those who understand its meaning. We did not find evidence to either confirm or strongly refute the hypothesis that the left mid-fusiform gyrus responds *selectively* to visual written word forms (Cohen and Dehaene, 2004). However, our data could be argued to support an amended version of this hypothesis: that the VWFA is involved in the integration of orthographically structured input with visual word form representations, regardless of input form.

A broader account of our data suggests that the left mid-fusiform gyrus may play a role in mapping between the perception of meaningful, or potentially meaningful, stimuli and higher-order representations, not only orthographic, but also phonological and semantic (Devlin et al., 2006; Xue et al., 2006). The current data extend this account of the mid-fusiform gyrus to include not only static but also dynamic visual stimuli. This interpretation is based on post-hoc suggestions regarding the levels of representation with which our different stimuli may integrate. To test this hypothesis, further studies in which these mappings are explicitly weighted by manipulating both stimuli and task requirements are necessary. Nevertheless, the present study adds a unique perspective on the role of this controversial region of the brain and on the neural systems supporting fingerspelling perception in deaf native signers.

## Figures and Tables

**Figure S1:**
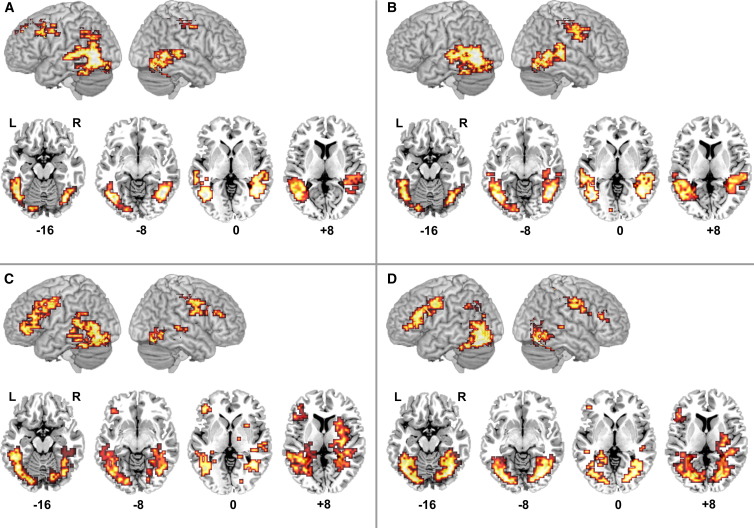
Activation for each visual input form relative to the baseline task in hearing non-signers (*n* = 13; voxelwise *P* = 0.05; clusterwise *P* = 0.0025). [A] FS > baseline; [B] SL > baseline; [C] TEXT > baseline; [D] PICS > baseline. On lateral renderings, activated voxels up to 15 mm beneath the cortical surface are displayed. Contiguous axial slices from TC *z* = − 16 to 8 mm are also shown. L = left. R = right.

**Table 1:** Participant characteristics: mean [S.D.] of age, non-verbal IQ percentile, reading age, and productive English vocabulary

	Age	NVIQ percentile	Reading age	Vocabulary (max = 30)
Deaf (*n* = 13)	27.4 [93.1 months] range: 227–591 months	88.2 [13.3] range: 50–98	15.11 [34.1 months] range: 135–246 months	28.0 [2.61] range: 22–30
Hearing (*n* = 13)	29.4 [73.8 months] range: 227–520 months	83.2 [19.6] range: 25–99	19.09 [21.7 months] range: 200–264 months	29.4 [0.96] range: 27–30

Deaf and hearing participants did not differ in age or NVIQ (*P*-values > 0.1). However, reading and vocabulary scores were higher in hearing than deaf participants (*P*-values < 0.025).

**Table 2:** Mean (*mean %*; [S.D.]) accuracy (Acc.; max = 5, except for baseline task: max = 20) and reaction time (RT in ms; [S.D.]) across all four visual input forms and the baseline task for deaf and hearing participants

	FS		SL		TEXT		PICS		Baseline	
Acc.	RT	Acc.	RT	Acc.	RT	Acc.	RT	Acc.	RT
Deaf	4.5	2219	4.8	1498	4.9	706	4.8	653	19.7	463
*n* = 13	*90%*		*96%*		*98%*		*96%*		*98.5%*	
	[.52]	[56]	[.44]	[25]	[.28]	[16]	[.44]	[149]	[.86]	[88]
Hearing	–	–	–	–	4.9	696	5.0	582	19.9	464
*n* = 13	–		–		*98%*		*100%*		*99.5%*	
	[–]	[–]	[–]	[–]	[.33]	[170]	[0]	[79]	[.38]	[58]

FS = fingerspelled English words; SL = BSL signs; TEXT = written English words; PICS = pictures.

**Table 3:** Activation for each visual input form relative to the baseline task in deaf native signers (*n* = 13)

	Size	*x*	*y*	*z*	BA
voxels/cm^3^
*Fingerspelled English words (FS)*					
L middle occipital/inferior temporal gyri*^,^^a^	1298/350.46	− 47	− 63	− 7	*19/37*
* Anterior cingulate*	*154/41.58*	*0*	*15*	*46*	*6*
* L precentral gyrus*	*371/100.17*	*− 43*	−* 4*	*46*	*6*
* L inferior/middle/superior frontal gyri*	*196/52.92*	*− 43*	*37*	*10*	*46*
* L inferior/middle temporal gyri*	*142/38.34*	*− 47*	−* 41*	*3*	*22*
* L superior temporal gyrus*	*222/59.94*	*− 58*	−* 26*	*10*	*41*
L fusiform gyrus*	*213/57.51*	*− 47*	−* 63*	−* 7*	*19/37*
R inferior/middle temporal gyri*	290/78.30	43	− 59	0	37
L precuneus	201/54.27	− 29	− 44	43	7
R inferior/middle frontal gyri	241/65.07	40	22	20	45/46

*BSL signs (SL)*					
L middle occipital/inferior temporal gyri*	479/129.33	− 47	− 63	− 7	19/37
R fusiform/inferior temporal gyri*	321/86.67	43	− 56	− 10	37
L middle frontal gyrus	428/115.56	− 40	19	26	9
R middle frontal gyrus	160/43.20	47	0	40	6

*Written English words (TEXT)*					
L fusiform gyrus**	185/49.95	− 22	− 81	− 10	19
L middle frontal gyrus	246/66.42	− 40	19	26	9

*Pictures (PICS)*					
L fusiform gyrus/inferior temporal gyri*	503/135.81	− 43	− 63	− 10	19/37
R fusiform gyrus/inferior temporal gyri**	295/79.65	36	− 63	− 20	20/36
L inferior/middle frontal gyri	170/45.90	− 36	19	23	44/46

Coordinates report maxima of 3-D clusters. L = left. R = right. Voxelwise *P* = 0.05; clusterwise *P* = 0.0025. * Indicates focal coordinates consistent with the VWFA (or its right hemisphere homologue). ** Indicates clusters in which there are subpeaks consistent with the VWFA (or its right hemisphere homologue). ^a^ This cluster has been decomposed into six sub-clusters using a model-driven cluster analysis (MClust; see Materials and methods section). Details for each of the six sub-clusters revealed by MClust appear below the peak cluster, indented and in italics.

**Table 4:** Activation for each visual input form relative to the baseline task in hearing non-signers (*n* = 13)

	Size	*x*	*y*	*z*	BA
voxels/cm^3^
*Fingerspelled English words (FS)*					
L inferior temporal gyrus*	521/140.67	− 43	− 67	0	37
R middle occipital/inferior temporal gyri*	304/82.08	47	− 59	− 7	19/37
R inferior parietal lobule	162/43.74	33	− 37	43	40
L superior frontal gyrus	203/54.81	− 11	44	40	8

*BSL signs (SL)*					
L middle occipital/inferior temporal gyri*	534/144.18	− 43	− 67	− 3	19/37
R middle occipital/inferior temporal gyri*	365/98.55	43	− 59	− 3	19/37
R postcentral gyrus	254/68.58	43	− 19	46	1

*Written English words (TEXT)*					
L fusiform gyrus*	603/162.81	− 40	− 67	− 10	19
R fusiform gyrus**	457/123.39	33	− 70	− 10	19
L precentral/inferior frontal gyri	878/237.06	− 36	4	33	6/44

*Pictures (PICS)*					
L fusiform gyrus**	2506/676.62	− 40	− 67	− 10	19

Coordinates report maxima of 3-D clusters. L = left. R = right. Voxelwise *P* = 0.05; clusterwise *P* = 0.0025. * Indicates focal coordinates consistent with the VWFA (or its right hemisphere homologue). ** Indicates clusters in which there are subpeaks consistent with the VWFA (or its right hemisphere homologue).

**Table 5:** Orthographic vs. non-orthographic static stimuli in deaf native signers and hearing non-signers (*n* = 26)

	Size	*x*	*y*	*z*	BA
voxels/cm^3^
*Main effect of visual input form: TEXT* > *PICS*					
L middle/superior temporal gyri	69/18.63	− 51	− 48	7	21/22

*Main effect of visual input form: PICS* > *TEXT*					
L middle occipital/fusiform gyri**	410/110.70	− 29	− 78	− 7	18/19
R cerebellum**	524/141.48	33	− 59	− 20	

Coordinates report maxima of 3-D clusters. L = left. R = right. TEXT = written English words. PICS = pictures. Voxelwise *P* = 0.05; clusterwise *P* = 0.005. ** Indicates clusters in which there are subpeaks consistent with the VWFA (or its right hemisphere homologue).

**Table 6:** Orthographic and non-orthographic visuo-dynamic stimuli in deaf native signers and hearing non-signers (*n* = 26)

	Size	*x*	*y*	*z*	BA
voxels/cm^3^
*Main effect of group: Deaf > Hearing*					
L middle occipital/inferior temporal gyri*	259/69.93	− 47	− 63	− 7	19/37
R fusiform/inferior temporal gyri*	122/32.94	43	− 56	− 10	37

*Main effect of Visual Input Form: FS > SL*					
L middle occipital/inferior temporal gyri*	129/34.83	− 47	− 63	− 7	19/37
R fusiform gyrus**	111/29.97	40	− 59	− 13	37
L inferior frontal gyrus	89/24.03	− 43	7	33	44

*Interaction*					
L inferior temporal gyrus*	97/26.19	− 43	− 67	0	37
R middle occipital/inferior temporal gyri*	123/33.21	43	− 59	− 3	19/37

Coordinates report maxima of 3-D clusters. L = left. R = right. FS = fingerspelled English words. SL = signed language (BSL). Voxelwise *P* = 0.05; clusterwise *P* = 0.005. * Indicates focal coordinates consistent with the VWFA (or its right hemisphere homologue). ** Indicates clusters in which there are subpeaks consistent with the VWFA (or its right hemisphere homologue).

**Table 7:** Activation greater for FS than SL [with reaction times to FS and SL stimuli included as a covariate] in deaf native signers (*n* = 13)

	Size	*x*	*y*	*z*	BA
voxels/cm^3^
L middle occipital/inferior temporal gyri*	214/57.78	− 43	− 59	− 3	19/37
[L middle occipital/inferior temporal gyri*]	[125/33.75]	[− 47]	[− 63]	[− 3]	[19/37]
R middle/inferior temporal gyrus**	108/29.16	43	− 56	3	19/37
[R middle/inferior temporal gyrus*]	[155/41.85]	[47]	[− 56]	[0]	[19/37]
L inferior frontal gyrus	102/27.54	− 43	7	33	44
[–]	[–]	[–]	[–]	[–]

Coordinates report maxima of 3-D clusters. L = left. R = right. Voxelwise *P* = 0.05; clusterwise *P* = 0.005. * Indicates focal coordinates consistent with the VWFA (or its right hemisphere homologue). ** Indicates clusters in which there are subpeaks consistent with the VWFA (or its right hemisphere homologue).

**Table 8:** Foci [and range] of greater activation for FS than SL perception in individual deaf native signers (*n* = 13) consistent with the VWFA (TC *x* = − 37 to − 48; TC *y* = − 43 to − 70; TC *z* = − 1 to − 17) and/or its right hemisphere homologue (TC *x* = 37 to 48; TC *y* = − 43 to − 70; TC *z* = − 1 to − 17)

	Left hemisphere				Right hemisphere			
Size (voxels/cm^3^)	*x*	*y*	*z*	Size (voxels/cm^3^)	*x*	*y*	*z*
01	134/36.18	− 47	(− 41)	− 17	35/9.45	47	− 52	− 3
		[− 36, − 51]	[− 26, − 63]	[0, − 23]		[36, 51]	[− 52, − 59]	[3, − 3]
02	104/28.08	− 43	− 67	− 10	94/25.38	40	− 67	− 10
		[− 32, − 47]	[− 44, − 81]	[3, − 23]		[11, 40]	[− 67, − 81]	[− 10, − 13]
03	114/30.78	− 43	− 59	− 3	312/84.24	43	− 48	− 7
		[− 36, − 51]	[− 59, − 78]	[0, − 23]		[32, 54]	[− 37, − 70]	[3, − 26]
04	50/13.50	− 47	− 63	− 7	49/13.23	47	− 44	(0)
		[− 40, − 58]	[− 48, − 67]	[0, − 16]		[43, 58]	[− 33, − 59]	[3, − 3]
05	–	–	–	–	–	–	–	–
06	107/28.89	− 47	− 70	(3)	74/19.98	43	− 56	(0)
		[− 36, − 51]	[− 56, − 81]	[13, − 10]		[29, 43]	[− 30, − 70]	[7, − 10]
07	47/12.69	− 43	− 67	− 13	197/53.19	40	− 67	− 7
		[− 40, − 47]	[− 56, − 67]	[− 7, − 20]		[36, 61]	[− 33, − 74]	[13, − 23]
08	29/7.83	− 40	− 44	− 10	37/9.99	43	52	(3)
		[− 32, − 40]	[− 44, − 59]	[− 10, − 20]		[36, − 51]	[− 52, − 63]	[7, − 3]
09	45/12.15	(− 36)	− 63	− 13	49/13.23	(36)	− 59	− 7
		[− 36, − 54]	[− 56, − 67]	[3, − 16]		[36, 47]	[− 56, − 67]	[− 7, − 16]
10	–	–	–	–	230/62.10	43	− 63	− 10
						[36, 61]	[− 26, − 70]	[16, − 16]
11	62/16.74	− 43	− 70	− 17	125/33.75	47	− 63	− 13
		[− 36, − 47]	[− 56, − 70]	[− 3, − 20]		[7, 51]	[− 26, − 89]	[− 7, − 33]
12	47/12.69	− 40	− 63	(0)	62/16.74	(51)	− 56	− 3
		[− 40, − 51]	[− 63, − 67]	[7, − 10]		[47, 61]	[− 11, − 56]	[− 3, − 13]
13	–	–	–	–	32/8.64	(36)	− 67	− 17
						[36, 40]	[− 63, − 67]	[− 10, − 20]

Coordinates report maxima of 3-D clusters. Voxelwise *P* = 0.05; clusterwise *P* = 0.01. Peak coordinates in parentheses exceed the range of the VWFA by 1 to 4 mm.
