# Corrigendum to “Fingerspelling, signed language, text and picture processing in deaf native signers: The role of the mid-fusiform gyrus” [NeuroImage 35 (2007) 1287–1302]

**DOI:** 10.1016/j.neuroimage.2007.12.023

**Published:** 2008-04-01

**Authors:** Dafydd Waters, Ruth Campbell, Cheryl M. Capek, Bencie Woll, Anthony S. David, Philip K. McGuire, Michael J. Brammer, Mairéad MacSweeney

**Affiliations:** a Behavioural and Brain Sciences Unit, Institute of Child Health, University College London, 30 Guilford Street, London WC1N 1EH, UK; b Deafness, Cognition and Language Research Centre, Department of Human Communication Science, University College London, 49 Gordon Square, London WC1H 0PD, UK; c Institute of Psychiatry, King's College London, De Crespigny Park, London SE5 8AF, UK

We recently noticed an error in the Methods section of this paper. Voxel size in Talairach space was reported as 3 × 3 × 3 mm. It was actually 3.3 × 3.3 × 3.3 mm. Due to this error, and to a systematic error in calculating metric volume, activation volumes were reported incorrectly throughout the paper. These errors have no impact, however, on the arguments presented.

In the Results section, and in Table 3, Table 4, Table 5, Table 6, Table 7, Table 8, activation sizes are given as both number of activated voxels and metric volume. In all cases, the figure reported for the number of voxels is correct, whereas the volume reported is incorrect.

**Table 3:** Metric volume corrected: Activation for each visual input form relative to the baseline task in deaf native signers (*n* = 13)

	Size (voxels/cm^3^)	*x*	*y*	*z*	BA
Fingerspelled English words (FS)					
L middle occipital/inferior temporal gyri^a^^,^^b^	1298/46.65	− 47	− 63	− 7	19/37
*Anterior cingulate*	*154/5.53*	*0*	*15*	*46*	*6*
*L precentral gyrus*	*371/13.33*	*− 43*	*− 4*	*46*	*6*
*L inferior/middle/superior frontal gyri*	*196/7.04*	*− 43*	*37*	*10*	*46*
*L inferior/middle temporal gyri*	*142/5.10*	*− 47*	*− 41*	*3*	*22*
*L superior temporal gyrus*	*222/7.98*	*− 58*	*− 26*	*10*	*41*
*L fusiform gyrus*^a^	*213/7.65*	*− 47*	*− 63*	*− 7*	*19/37*
R inferior/middle temporal gyri^a^	290/10.42	43	− 59	0	37
L precuneus	201/7.22	− 29	− 44	43	7
R inferior/middle frontal gyri	241/8.66	40	22	20	45/46
BSL signs (SL)					
L middle occipital/inferior temporal gyri^a^	479/17.21	− 47	− 63	− 7	19/37
R fusiform/inferior temporal gyri^a^	321/11.54	43	− 56	− 10	37
L middle frontal gyrus	428/15.38	− 40	19	26	9
R middle frontal gyrus	160/5.75	47	0	40	6
Written English words (TEXT)					
L fusiform gyrus^c^	185/6.65	− 22	− 81	− 10	19
L middle frontal gyrus	246/8.84	− 40	19	26	9
Pictures (PICS)					
L fusiform gyrus/inferior temporal gyri^a^	503/18.08	− 43	− 63	− 10	19/37
R fusiform gyrus/inferior temporal gyri^c^	295/10.60	36	− 63	− 20	20/36
L inferior/middle frontal gyri	170/6.11	− 36	19	23	44/46

Coordinates report maxima of 3-D clusters.

L = left. R = right. Voxelwise *P* = 0.05. Clusterwise *P* = 0.0025.

a Indicates that focal coordinates consistent with the VWFA (or its right hemisphere homologue).

b This cluster has been decomposed into six sub-clusters using a model-driven cluster analysis (MClust; see Methods section). Details for each of the six sub-clusters revealed by MClust appear below the peak cluster, indented and in italics.

c Indicates clusters in which there are subpeaks consistent with the VWFA (or its right hemisphere homologue).

**Table 4:** Metric volume corrected: Activation for each visual input form relative to the baseline task in hearing non-signers (*n* = 13)

	Size (voxels/cm^3^)	*x*	*y*	*z*	BA
Fingerspelled English words (FS)					
L inferior temporal gyrus^a^	521/18.72	− 43	− 67	0	37
R middle occipital/inferior temporal gyri^a^	304/10.92	47	− 59	− 7	19/37
R inferior parietal lobule	162/5.82	33	− 37	43	40
L superior frontal gyrus	203/7.30	− 11	44	40	8
BSL signs (SL)					
L middle occipital/inferior temporal gyri^a^	534/19.19	− 43	− 67	− 3	19/37
R middle occipital/inferior temporal gyri^a^	365/13.12	43	− 59	− 3	19/37
R postcentral gyrus	254/9.13	43	− 19	46	1
Written English words (TEXT)					
L fusiform gyrus^a^	603/21.67	− 40	− 67	− 10	19
R fusiform gyrus^b^	457/16.42	33	− 70	− 10	19
L precentral/inferior frontal gyri	878/31.55	− 36	4	33	6/44
Pictures (PICS)					
L fusiform gyrus^b^	2506/90.06	− 40	− 67	− 10	19

Coordinates report maxima of 3-D clusters.

L = left. R = right. Voxelwise *P* = 0.05. Clusterwise *P* = 0.0025.

a Indicates focal coordinates consistent with the VWFA (or its right hemisphere homologue).

b Indicates clusters in which there are subpeaks consistent with the VWFA (or its right hemisphere homologue).

**Table 5:** Metric volume corrected: Orthographic vs. non-orthographic static stimuli in deaf native signers and hearing non-signers (*n* = 26)

	Size (voxels/cm^3^)	*x*	*y*	*z*	BA
Main effect of visual input form: TEXT > PICS					
L middle/superior temporal gyri	69/2.48	− 51	− 48	7	21/22
Main effect of visual input form: PICS > TEXT					
L middle occipital/fusiform gyri^a^	410/14.73	− 29	− 78	− 7	18/19
R cerebellum^a^	524/18.83	33	− 59	− 20	

Coordinates report maxima of 3-D clusters.

L = left. R = right. TEXT = written English words. PICS = pictures. Voxelwise *P* = 0.05. Clusterwise *P* = 0.005.

a Indicates clusters in which there are subpeaks consistent with the VWFA (or its right hemisphere homologue).

**Table 6:** Metric volume corrected: Orthographic and non-orthographic visuo-dynamic stimuli in deaf native signers and hearing non-signers (*n* = 26)

	Size (voxels/cm^3^)	*x*	*y*	*z*	BA
Main effect of group: Signers > Non-signers					
L middle occipital/inferior temporal gyri^a^	259/9.31	− 47	− 63	− 7	19/37
R fusiform/inferior temporal gyri^a^	122/4.38	43	− 56	− 10	37
Main effect of visual input form: FS > SL					
L middle occipital/inferior temporal gyri^a^	129/4.64	− 47	− 63	− 7	19/37
R fusiform gyrus^b^	111/3.99	40	− 59	− 13	37
L inferior frontal gyrus	89/3.20	− 43	7	33	44
Interaction					
L inferior temporal gyrus^a^	97/3.49	− 43	− 67	0	37
R middle occipital/inferior temporal gyri^a^	123/4.42	43	− 59	− 3	19/37

Coordinates report maxima of 3-D clusters.

L = left. R = right. FS = fingerspelled English words. SL = signed language (BSL). Voxelwise *P* = 0.05. Clusterwise *P* = 0.005.

a Indicates focal coordinates consistent with the VWFA (or its right hemisphere homologue).

b Indicates clusters in which there are subpeaks consistent with the VWFA (or its right hemisphere homologue).

**Table 7:** Metric volume corrected: Activation greater for FS than SL [with reaction times to FS and SL stimuli included as a covariate] in deaf native signers (*n* = 13)

	Size (voxels/cm^3^)	*x*	*y*	*z*	BA
L middle occipital/inferior temporal gyri^a^	214/7.69	− 43	− 59	−3	19/37
[L middle occipital/inferior temporal gyri^a^]	[125/4.49]	[− 47]	[− 63]	[− 3]	[19/37]
R middle/inferior temporal gyrus^b^	108/3.88	43	− 56	3	19/37
[R middle/inferior temporal gyrus^a^]	[155/5.57]	[47]	[− 56]	[0]	[19/37]
L inferior frontal gyrus	102/3.67	− 43	7	33	44
[–]	[–]	[–]	[–]	[–]	[–]

Coordinates report maxima of 3-D clusters.

L = left. R = right. Voxelwise *P* = 0.05. Clusterwise *P* = 0.005.

a Indicates focal coordinates consistent with the VWFA (or its right hemisphere homologue).

b Indicates clusters in which there are subpeaks consistent with the VWFA (or its right hemisphere homologue).

**Table 8:** Metric volume corrected: Foci [and range] of greater activation for FS than SL perception in individual deaf native signers (*n* = 13) consistent with the VWFA (TC: *x* = − 37 to − 48; *y* = − 43 to − 70; *z* = − 1 to − 17) and/or its right hemisphere homologue (TC: *x*= 37 to 48; *y* = − 43 to − 70; *z* = − 1 to − 17)

	Left hemisphere				Right hemisphere			
	Size (voxels/cm^3^)	*x*	*y*	*z*	Size (voxels/cm^3^)	*x*	*y*	*z*
01	134/4.82	− 47	(− 41)	− 17	35/1.26	47	− 52	− 3
		[− 36, − 51]	[− 26, − 63]	[0, − 23]		[36, 51]	[− 52, − 59]	[3, − 3]
02	104/3.74	− 43	− 67	− 10	94/3.38	40	− 67	− 10
		[− 32, − 47]	[− 44, − 81]	[3, − 23]		[11, 40]	[− 67, − 81]	[− 10, − 13]
03	114/4.10	− 43	− 59	− 3	312/11.21	43	− 48	− 7
		[− 36, − 51]	[− 59, − 78]	[0, − 23]		[32, 54]	[− 37, − 70]	[3, − 26]
04	50/1.80	− 47	− 63	− 7	49/1.76	47	− 44	(0)
		[− 40, − 58]	[− 48, − 67]	[0, − 16]		[43, 58]	[− 33, − 59]	[3, −3]
05	–	–	–	–	–	–	–	–
06	107/3.85	− 47	− 70	(3)	74/2.66	43	− 56	(0)
		[− 36, − 51]	[− 56, − 81]	[13, − 10]		[29, 43]	[− 30, - 70]	[7, − 10]
07	47/1.69	− 43	− 67	− 13	197/7.08	40	− 67	− 7
		[− 40, − 47]	[− 56, − 67]	[− 7, − 20]		[36, 61]	[− 33, − 74]	[13, − 23]
08	29/1.04	− 40	− 44	− 10	37/1.33	43	− 52	(3)
		[− 32, − 40]	[− 44, − 59]	[− 10, − 20]		[36, 51]	[− 52, − 63]	[7, − 3]
09	45/1.62	(− 36)	− 63	− 13	49/1.76	(36)	− 59	− 7
		[− 36, − 54]	[− 56, − 67]	[3, − 16]		[36, 47]	[− 56, − 67]	[− 7, − 16]
10	–	–	–	–	230/8.27	43	− 63	− 10
						[36, 61]	[− 26, − 70]	[16, − 16]
11	62/2.23	− 43	− 70	− 17	125/4.49	47	− 63	− 13
		[− 36, − 47]	[− 56, − 70]	[− 3, − 20]		[7, 51]	[− 26, − 89]	[− 7, − 33]
12	47/1.69	− 40	− 63	(0)	62/2.23	(51)	− 56	− 3
		[− 40, − 51]	[− 63, − 67]	[7, − 10]		[47, 61]	[− 11, − 56]	[− 3, − 13]
13	–	–	–	–	32/1.15	(36)	− 67	− 17
						[36, 40]	[− 63, − 67]	[− 10, −20]

Coordinates report maxima of 3-D clusters.

Voxelwise *P* = 0.05. Clusterwise *P* = 0.01.

Peak coordinates in parentheses exceed the range of the VWFA by 1 to 4 mm.

The following formula may be used to calculate the correct volume for these activations:

$$[\text{number of voxels}]\times [\text{voxel size in Talairach space}\;(3.3\times 3.3\times 3.3\;\text{mm}=35.937\;{\text{mm}}^{3})]/1000={\text{cm}}^{3}$$

Corrected tables have been reproduced here for the reader's convenience.

